# Defining how microorganisms benefit human health

**DOI:** 10.1111/1751-7915.13685

**Published:** 2020-10-25

**Authors:** Maria L. Marco

**Affiliations:** ^1^ Department of Food Science and Technology University of California, Davis Davis CA 95616 USA

## Abstract

An appreciation for how microorganisms can benefit human health has grown over the past century. The future of this research will be to identify the specific microbial enzymatic pathways and molecules necessary for health promotion. Some of these ‘beneficial factors’ are already known for probiotics and species in the human microbiome, however, precise descriptions of the mechanistic details for their effects remain to be discovered. The need for this research is elevated by the potential use of microorganisms for preventing and treating the non‐communicable diseases which are now the leading causes of death worldwide.

The ‘golden age of bacteriology’ was the most impactful and long‐lasting period of discovery in the history of microbiological research (Blevins and Bronze, 2010). Initiated by the work of Louis Pasteur in the 1860s, Pasteur, Robert Koch, and other contemporaries disproved spontaneous generation, verified the germ theory of disease, identified many of the known bacterial pathogens, and developed microbiology laboratory methods still in use today. As with many scientific breakthroughs, advances made by the 19th century microbiologists were stimulated by prevailing societal needs. At that time, infectious disease was the primary cause of death (Smith *et al*., 2012). Pneumonia, tuberculosis, and diarrhoea were at the top of all causes of mortality (Department of Commerce and Labor, Bureau of the Census, 1906). The work of those early microbiologists resulted in the development of public health programs and widespread changes in societal norms and attitudes that emphasized how to minimize exposure to or eliminate ‘germs’ (Tomes, 1998; Smith *et al*., 2012).

We are now in a new age in microbiological research (Blaser, 2014). The start of this era is marked by the work of Carl Woese and colleagues who pioneered the development of methods to study microbial phylogeny and identify and investigate microorganisms without the need for laboratory cultivation (Pace, 2009). Subsequent advancements, most notably improvements in DNA sequencing technologies and computing power enabled broad surveys and deep interrogation of microbial habitats (Escobar‐Zepeda *et al*., 2015). Much like the catalyst for the rapid progress that occurred in the late 1800s, the present time also has societal urgency. Cardiovascular diseases and other non‐communicable diseases are now the leading causes of death worldwide (GBD 2016 Causes of Death Collaborators, 2017). Climate change and other human‐made damage to fragile ecosystems are existential threats. Concurrent with the emergence of these issues, focus has shifted away from emphasis on microorganisms as germs to also consider how they can be beneficial and useful for human health and the environment. Expansion in this direction of inquiry involves all aspects of microbiology including asking how microorganisms may be used for preventing or reversing pollution, improving food security and safety, and maintaining human health and well‐being. Although research over the past 100 years has identified microorganisms able to confer benefits, there still remain significant gaps on the precise molecular mechanisms responsible for those outcomes.

Knowledge on how specific microbial compounds and activities result in health benefits has been developing over a long trajectory (Yong, 2016). Commensal microorganisms were understood to inhabit the human body since the time of Antonie van Leeuwenhoek. At the start of the twentieth century, Issac Kendall described the gut as a ‘singularly perfect incubator’ (Kendall, 1909) and Elie Metchnikoff and Henry Tissier proposed applying microorganisms in fermented foods or from the gastrointestinal tract to support human health (McFarland, 2015). Throughout the twentieth century, the science of probiotics continued to develop (McFarland, 2015) alongside advancements in deciphering the physiology and function of commensal microbial inhabitants of the human body (Schwiertz, 2016). It is now firmly established that there are positive roles for the microbiota inhabiting the alimentary, respiratory, and vaginal tracts, skin, and other exposed sites (Cho and Blaser, 2012). Systematic reviews and meta‐analyses of clinical trials support the use of probiotics (Merenstein *et al*., 2020). To address questions of how microbes can be beneficial, numerous general mechanisms have been proposed such as prevention of pathogen colonization, modulation of the immune system, the digestion, detoxification, and production of nutrients, stimulation of cellular differentiation, improvement of barrier function, and alteration of the gut‐brain axis. Those broad mechanistic categories have been used to summarize potential benefits conveyed by the autochthonous microbiota and probiotics alike.

The future of this research is the identification of the specific metabolites, proteins, and other compounds made by microorganisms that trigger specific cellular responses in the host to result in sustaining or improving health and well‐being. This work will lead to precise mechanistic descriptions of ‘beneficial factors’, or the specific microbial enzymatic pathways and molecules necessary for health promotion. As might be expected based on the known complexity of the human microbiome, it will not be a trivial task. Similar to the massive prior and ongoing efforts to identify virulence factors of human pathogens and molecular pathogenesis mechanisms that lead to disease, so too will there be the need for allocation of significant effort and resources to this still emerging field of study.

Presently, only a fraction of beneficial, or functional, factors made by microorganisms are known. Short‐chain fatty acids (SCFA) are currently the best characterized microbial metabolites regarded to benefit health. These compounds are major products of anaerobic carbohydrate and protein fermentation by intestinal microorganisms. Enzymatic pathways for SCFA acetate, propionate, and butyrate biosynthesis are known (Louis and Flint, 2017), as are the SCFA receptors (free fatty acid/G protein‐coupled receptors FFA3/GPR41 and FFA2/GPR43) that result in modulation of metabolic, immune, and endocrine responses (Bolognini *et al*., 2021). Other microbial compounds generated as intermediate or end products of microbial metabolism were shown to benefit health, although a more complete description of mechanistic details for their effects remains to be discovered. Examples include metabolites of amino acids such as tryptophan, glutamate, histidine, and phenylalanine which are modified by some bacteria to compounds with neuroactive or immunomodulatory properties (Engevik and Versalovic, 2017; Peters *et al*., 2019). Lactic acid, a compound present in high abundance in some fermented foods, downregulates pro‐inflammatory responses and stimulates intestinal development (Iraporda *et al*., 2015; Lee *et al*., 2018). Vitamins including folates, riboflavin, cobalamin and vitamin K are synthesized for use by microorganisms but may also be absorbed in the digestive tract (Ruan *et al*., 2020), and certain chromosomal CpG DNA motifs are immunomodulatory (Li *et al*., 2020). Lastly, overall growth of certain microorganisms may be beneficial by resulting in competitive exclusion of pathogenic microbes via the utilization of scarce resources (for example iron acquisition in the digestive tract (Deriu *et al*., 2013)).

Secondary metabolites and signalling molecules are also capable of supporting human health. These functional factors include compounds such as bacteriocins that are best understood for their bactericidal activity against human pathogens but also confer direct effects on tissues with potential use as anticancer and barrier protective agents (Hegarty *et al*., 2016; Heeney *et al*., 2019). Products of nonribosomal peptide synthetases also have antimicrobial (Engevik and Versalovic, 2017) or other bioactive (Guo *et al*., 2017) properties and are represented in the many small‐molecule gene clusters present in the human microbiome (Cimermancic *et al*., 2014; Donia *et al*., 2014). Quorum‐sensing peptides and different peptidic compounds induce cytoprotective responses (Tao *et al*., 2006; Fujiya *et al*., 2007). Other secreted structures such as extracellular membrane vesicles can carry a variety of compounds and may result in anti‐inflammatory, neurotrophic and other effects (Caruana and Walper, 2020).

Microbial cell surface compounds are also recognized by epithelial and immune cells. Compounds with microbial associated microbial patterns that induce innate immunity such as those found in peptidoglycan, lipoteichoic acids, flagella, and pili are made by probiotics and human commensals (Lebeer *et al*., 2010; Liu *et al*., 2020). Certain cell surface and membrane proteins confer distinct host cell responses including disease mitigation and epithelial protection (Engevik and Versalovic, 2017; Plovier *et al*., 2017; Liu *et al*., 2020; Yan and Polk, 2020). Even some membrane lipids such as sphingolipids (An *et al*., 2014) and those synthesized as a result microbial detoxification polyunsaturated fatty acids (Miyamoto *et al*., 2019) may be beneficial.

Much of the evidence on health‐supporting microbial compounds is from studies on *Lactobacillus* and *Bifidobacterium*. Besides molecular characterization those general stemming from their use as probiotics (Lebeer *et al*., 2018), mechanistic studies of intestinal microbiota have shown that compounds made by Lactobacillus and *Bifidobacterium* are important for healthy gut function (Bottacini *et al*., 2017; Heeney *et al*., 2017). Lactobacilli are also highly abundant in the vagina (Ma *et al*., 2020) and are found in the upper respiratory tract (De Boeck *et al*., 2020). *Lactobacillus* and other lactic acid bacteria in fermented foods are sources of bacteria in the human gut microbiome (Pasolli *et al*., 2020; Taylor *et al*., 2020). These bacteria and products made as a result of their transformation of food components are important contributors to the health benefits resulting from the consumption of fermented foods (Marco *et al*., 2017). *Lactobacillus* and *Bifidobacterium* species will continue to be studied due to their presence in the human microbiome and regular intake in human diets. They are also efficacious in clinical studies, relatively easily applied (generally recognized as safe), amenable to genetic manipulation for mechanistic research, and there is already relatively abundant, albeit incomplete, knowledge on their genetic diversity, ecology and metabolism.

As the number of verified microorganism‐derived, health‐promoting compounds increases, so too will the diversity of microbial species capable of making them. Strains of *Escherichia coli, Bacillus, Propinibacterium* and *Saccharomyces boulardii* are currently used as probiotics. *Cutibacterium acnes* (Paetzold *et al*., 2019) and *Lactococcus lactis* (Radaic *et al*., 2020) have shown promise as probiotics on the skin and in the oral cavity, respectively. Among prominent bacterial species in the human intestine, pathways for polysaccharide metabolism by *Ruminococcus, Bacteroides* and other taxa will continue to be elucidated (Martens *et al*., 2014). *Faecalibacterium prausnitzii* and *Akkermansia mucinophila* have emerged as gut commensals with therapeutic properties and for which specific functional compounds have been identified (Quévrain *et al*., 2016; Plovier *et al*., 2017). Just as the immune system recognizes specific bacterial species (Belkaid and Harrison, 2017), it is expected that these examples are just the start to the identification of the microorganisms and compounds responsible for health promotion by the human microbiome.

Importantly, many of the known microbial beneficial factors were found as a result of prediction or educated guesses rather than through genetic or biochemical screening approaches. Identification of new compounds will be accelerated by expanding the use of comparative genomics, mutant libraries and biochemical characterization of cell fractions in combination with functional screens with reporter cell lines. However, it is noted that there are also limitations to those methods such as the need for genetically distinguishable features and the necessity for the beneficial compounds to be made in laboratory culture medium *in vitro*. The fact that some of these compounds will be essential and required for microbial growth and survival also creates challenges for identification with mutant libraries. Nonetheless, these global efforts are expected to be productive for identification of novel secondary metabolites and certain non‐essential, cell surface proteins or cell structures, many of which are likely to exhibit a high level of genetic variation between strains.

It is also expected that some beneficial factors will be widespread and shared among numerous microbial taxonomic groups. These compounds will include essential proteins and other fundamental components of microbial cells as well as those that arose during adaptation to selection pressures in the host and other environments. For example, the variety of bacterial species and enzymes responsible for the breakdown complex polysaccharides is expanding and showing some generalizable features (Flint *et al*., 2012; Cantu‐Jungles and Hamaker, 2020). For probiotics, there is an emerging view that well‐studied species known to confer health benefits may do so via the principle of ‘shared benefits’ (Sanders *et al*., 2018). This principle is based on the knowledge that certain bacterial species have conserved, or core, properties which may be responsible for improving health.

Once a more complete view of the functional metabolites and cell components made by microorganisms is known, it will be possible to propose personalized approaches, taking into account differences in human genetics, lifestyle, age and diet. It is already known that the composition of the gut microbiome is tightly linked to these factors (Lozupone *et al*., 2012). It is also established that probiotic cell composition changes *in situ* depending on diet and the prevailing conditions in the digestive tract (Marco and Tachon, 2013). However, until a more complete understanding of beneficial microbes is reached, diet, age, health and other characteristics should be collected in microbiome and probiotic intervention studies. These data may eventually be useful for who is most likely to be responsive to microbial treatment.

In the process of elucidating how individual microbial compounds improve health, research will move beyond correlation and associative studies to those that establish causation (Fischbach, 2018). Importantly, direct effects on epithelial, immune, and other cell types will need to be untangled from those resulting from modulating the function of the resident microbiome. Thus, gnotobiotic animal models and cell, tissue and organ cultures will continue to be needed and used in concert with animal models of health and disease. Besides focus on the properties of individual strains in isolation, advances in metagenome sequencing and bioinformatics methods are expected to continue to improve strain‐level resolution of the human microbiota (Yan *et al*., 2020). Systems biology approaches will be important for identifying the key features necessary for health promotion by addressing the complexity of the human microbiome and its capacity to synthesize thousands of metabolites (Greenblum *et al*., 2013). These findings can then be applied in model microbial community reconstructions (Toju *et al*., 2020). Identification of human cell receptors and signal transduction pathways modulated by the beneficial effector compounds will also be aided by the on‐going, rapid gains in knowledge in diverse fields such as mucosal immunology and endocrinology.

Ultimately, beneficial factors should be tested in purified forms to verify specificity and selectivity. The compounds should be given in doses needed for verifying physiologic effects and for confirming whether resident or probiotic microbes synthesize them in situ in quantities sufficient for the expected health outcomes. Rather than in a purified form, it may also be found that some beneficial microbial cell products are most effective when delivered by the microorganism itself. Intact microbial cells expressing multiple beneficial factors may be important for complementary or synergistic interactions with human tissues that may not be achieved when those compounds are provided separately.

When purified compounds and intact strains are tested, these efforts will ultimately improve probiotic applications by enabling the development of guidance on the specific dose, frequency and duration of strain application. In the future, strain use will be decided based on the production levels of the beneficial factors and site on the body where they are needed. Answers will also be reached on the need for temporary colonization as opposed to long‐term probiotic engraftment. Contraindications for probiotic use will be clearer as will the potential for adverse effects.

Identification of beneficial compounds and processes of autochthonous microbes is expected to lead to resolution to the overarching question of what defines a healthy human microbiome. Once this is known, new therapies and diets can be developed to adjust the numbers of the needed microorganisms to the appropriate levels. This knowledge will also be useful for improving interpretations of inter‐individual and inter‐study discrepancies between gut microbiome responses to drugs and individual dietary nutrients (e.g. resistant starch type 2 (Bendiks *et al*., 2020)).

Microbiology research in the late nineteenth century catalysed the development of medical and public health practices that led to significant reductions in infectious disease. A similar opportunity is upon us to apply the human microbiome and probiotics for preventing and treating non‐communicable, chronic conditions such as cardiovascular disease, obesity, type 2 diabetes and cancer. The urgency of this moment may provide the essential stimulus for accelerating multi‐disciplinary efforts to not only identify microorganisms required for good health and well‐being but also the compounds and underlying molecular mechanisms responsible for the benefits they confer.
